# Primary pancreatic lymphoma masquerading as pancreatic carcinoma: A case report and literature review

**DOI:** 10.1097/MD.0000000000045627

**Published:** 2025-10-31

**Authors:** Xingdong Xu, Tingting Zhang, Jinyu Liu, Xingzi Li, Gang Wang

**Affiliations:** a Department of Hepatobiliary Surgery, The First College of Clinical Medical Science, China Three Gorges University, Yichang Central People’s Hospital, Yichang, China; b Department of Radiology, The First College of Clinical Medical Science, China Three Gorges University, Yichang Central People’s Hospital, Yichang, China; c Department of Pathology, The First College of Clinical Medical Science, China Three Gorges University, Yichang Central People’s Hospital, Yichang, China.

**Keywords:** diagnosis, differential diagnosis, pancreatic lymphoma, R-CHOP regimen, surgery

## Abstract

**Rationale:**

Primary pancreatic lymphoma (PPL), representing 0.5% of pancreatic malignancies, poses diagnostic challenges due to overlapping clinical and imaging features with pancreatic adenocarcinoma. PPL is diagnosed by obtaining tissue samples via endoscopic ultrasound-guided fine-needle biopsy (EUS-FNB) or explorative laparotomy with biopsy. The R-CHOP regimen is the first-line chemotherapy protocol. This report presents a case initially misdiagnosed as pancreatic cancer, highlighting the critical role of histopathological confirmation.

**Patient concerns:**

A 55-year-old man presented with left upper abdominal pain for 20 days.

**Diagnoses:**

Computed tomography, magnetic resonance imaging, and fine needle biopsy suggested pancreatic cancer or neuroendocrine tumor. Postoperative pathology confirmed primary pancreatic lymphoma. The tumor was classified as CD20(+), BCL6(+), BCL2(+), and Ki-67(+, 60%).

**Interventions:**

The patient underwent laparoscopic distal pancreatectomy with splenectomy, followed by 6 cycles of R-CHOP chemotherapy.

**Outcomes:**

No recurrence was observed during a 2-year follow-up period.

**Lessons:**

This case highlights the diagnostic challenges associated with PPL and underscores the importance of histopathological confirmation for ambiguous pancreatic lesions. Although the role of surgery in localized disease requires further investigation, the R-CHOP regimen appears to be effective for managing PPL.

## 1. Introduction

Primary pancreatic lymphoma (PPL) is a rare extranodal non-Hodgkin lymphoma (NHL), constituting <0.5% of pancreatic malignancies and 0.6% to 0.7% of NHL cases.^[1]^ Over 80% of the cases are diffuse large B-cell lymphomas (DLBCL), predominantly affecting males aged 35 to 75 years.^[2]^ Clinical presentations, such as abdominal pain, weight loss, and jaundice mimic pancreatic adenocarcinoma, leading to frequent misdiagnosis. The definitive diagnosis of PPL is mainly obtained through tissue samples from endoscopic ultrasound (EUS)-guided biopsy or exploratory laparotomy biopsy.^[3]^ While the role of surgery in managing PPL is controversial, the R-CHOP regimen remains the first-line chemotherapy.

We report a surgically confirmed PPL case initially misdiagnosed as pancreatic adenocarcinoma or neuroendocrine tumor and synthesize current diagnostic and therapeutic paradigms.

## 2. Case report

A 55-year-old man was referred to the Department of Hepatobiliary Surgery with a 20-day history of left upper abdominal pain. The patient was in good condition, with no significant weight loss or medical history. Physical examination revealed no tenderness or rebound tenderness. Blood tests showed an elevated C-reactive protein level of 26.3 mg/L, while other parameters, including carcinoembryonic antigen and carbohydrate antigen 19-9 (CA19-9), were within normal limits.

**Figure 1.**
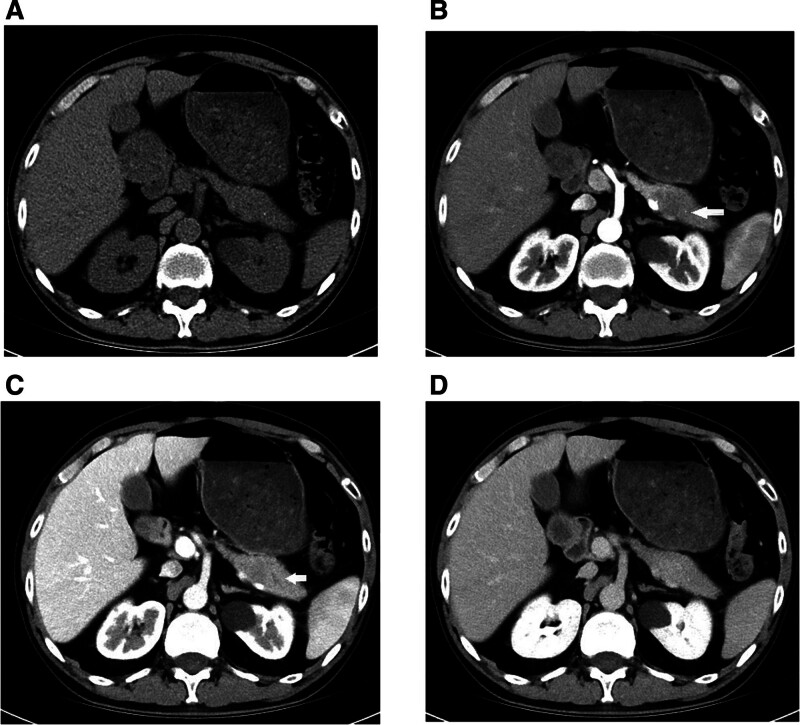
Contrast-enhanced abdominal CT images demonstrating a pancreatic tail mass (arrows) with gradual enhancement. (A) Non-contrast phase; (B) arterial phase; (C) portal venous phase; (D) delayed phase. CT = computed tomography.

**Figure 2.**
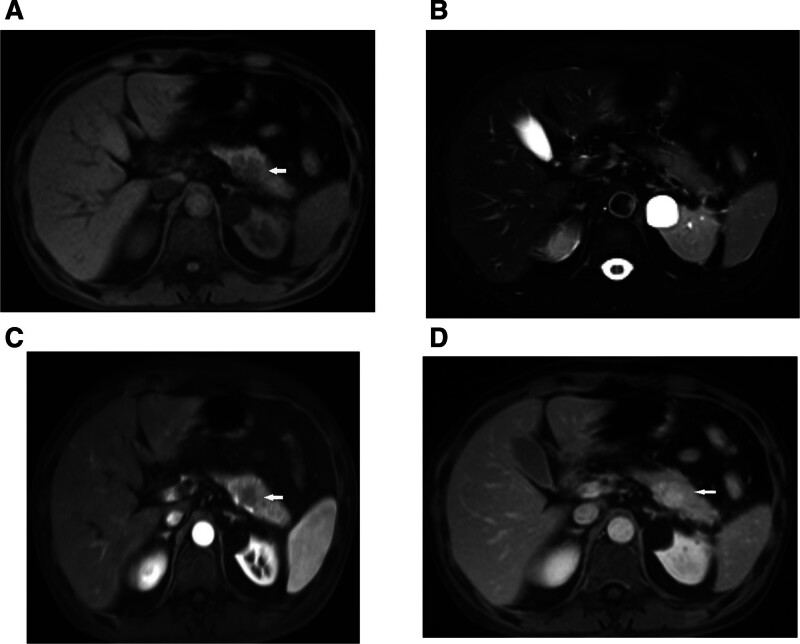
Magnetic resonance imaging (MRI) findings of the pancreatic tail mass. (A) T1-weighted image; (B) T2-weighted image; (C)arterial phase; (D) portal venous.

**Figure 3.**
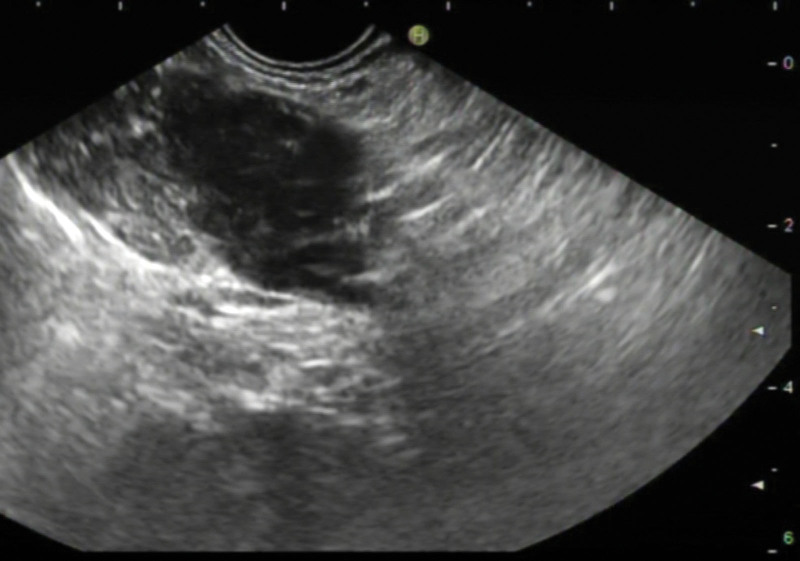
Endoscopic ultrasound (EUS) image. EUS reveals a well-defined, heterogeneous, and hypoechoic mass measuring 32 × 23 mm in the pancreatic tail. The overlying mucosal layers appear intact.

Abdominal contrast-enhanced computed tomography (CT) revealed a 30 × 19 mm hypodense mass in the pancreatic tail showing gradual enhancement (Fig. 1). Magnetic resonance imaging (MRI) and endoscopic ultrasound (EUS) identified a heterogeneous hypoechoic lesion without pancreatic duct dilation (Figs. 2–3). EUS-guided fine-needle biopsy (FNB) was performed using a 22-gauge needle with 3 passes; however, pathological examination of the samples revealed only fibrinoid necrosis with no identifiable tumor cells. Color Doppler ultrasonography of the abdominal cavity and superficial lymph nodes showed no abnormally enlarged lymph nodes.

**Figure 4.**
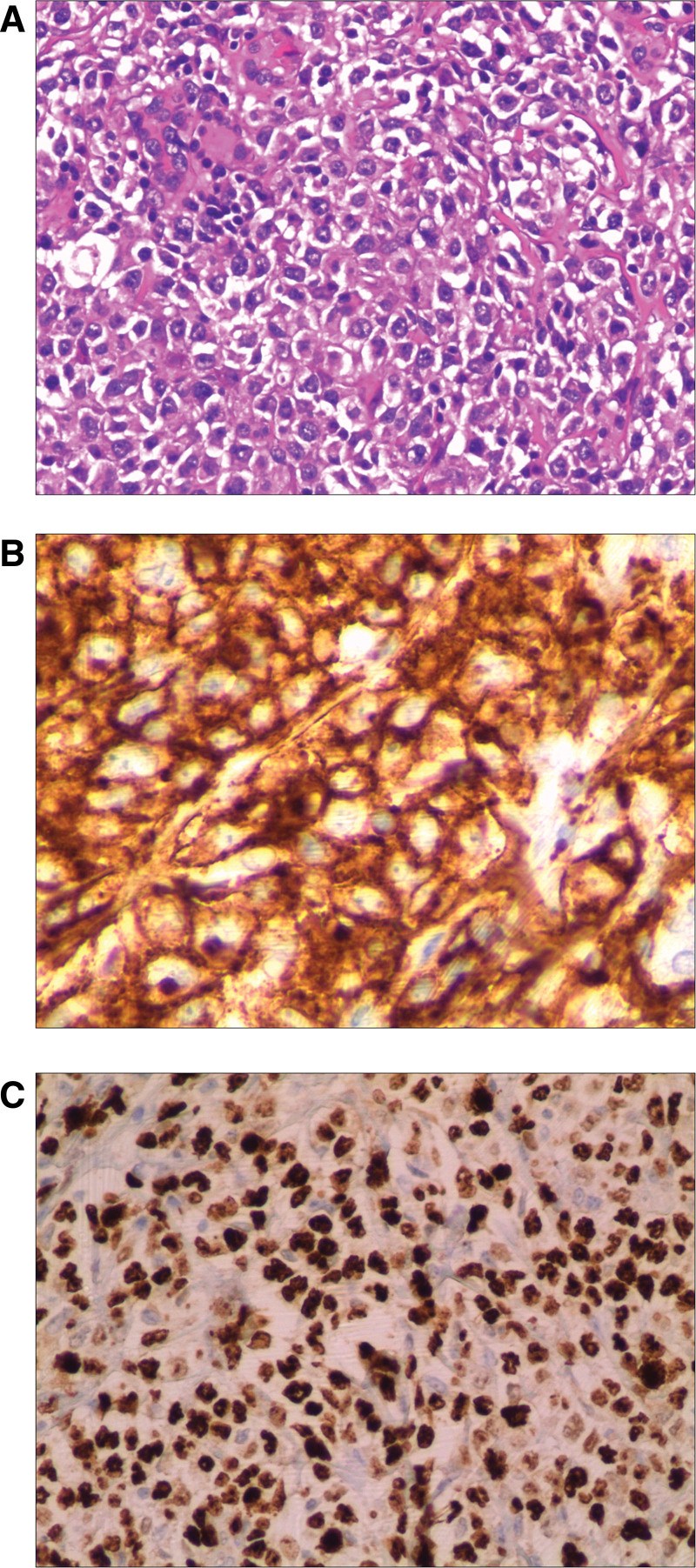
Histopathological and immunohistochemical analysis of the resected specimen. (A) Hematoxylin and eosin staining reveals diffuse infiltration by atypical lymphoid cells with irregular nuclear contours (×200). (B) Immunohistochemical staining is positive for CD20, confirming the B-cell lineage of the lymphoma (×400). (C) The Ki-67 proliferation index is high (approximately 60%), indicating a highly proliferative tumor(×200).

The initial diagnosis was pancreatic cancer or neuroendocrine tumor. The patient underwent laparoscopic distal pancreatectomy with splenectomy. Histological examination confirmed DLBCL with metastasis to the No. 11 lymph node group. Immunohistochemistry showed CD20(+), BCL6(+), BCL2(+), Ki-67 labeling index of 60% (Fig. 4). Following surgery, the patient received 6 cycles of R-CHOP chemotherapy (rituximab, cyclophosphamide, doxorubicin, vincristine, and prednisone), which he tolerated well. No clinical or radiological signs of recurrence were observed after a 2-year follow-up period.

## 3. Discussion

PPL is an exceedingly rare malignancy that accounts for only 1% of extranodal lymphomas.^[4]^ Due to nonspecific clinical symptoms and imaging features that overlap with pancreatic adenocarcinoma, PPL is frequently misdiagnosed, as exemplified in the present case

Currently, there are no specific biochemical markers for PPL.^[5]^ Peripheral blood white blood cell counts are usually normal and blood smears rarely reveal abnormalities. Unlike pancreatic adenocarcinoma, CA19-9 levels in patients with PPL are typically within the normal range unless biliary obstruction is present. Elevated lactate dehydrogenase levels, observed in 40% to 50% of PPL cases, may serve as a diagnostic clue, as this is uncommon in pancreatic cancer.^[6]^

CT and MRI are standard imaging modalities for evaluating pancreatic masses and diagnosing PPL. However, their limited specificity often hinders reliable differentiation between PPL and pancreatic adenocarcinoma. PPL is classified into 2 subtypes on CT: mass-forming (85–90% of cases) and diffuse infiltrative.^[4]^ The mass-forming subtype typically appears as a solitary lesion, most commonly in the pancreatic head (75–80%), with body or tail involvement being less common (<10%).^[7]^ Studies indicate that 70% of PPL lesions exceed 6 cm in diameter (40% >10 cm), contrasting sharply with pancreatic adenocarcinoma, where 60% of the tumors are < 6 cm.^[8]^ Despite their large size, PPL lesions rarely induce significant pancreatic duct dilatation due to their interstitial origin and soft consistency, which minimizes ductal compression. On contrast-enhanced CT, PPL exhibits a characteristic “progressive enhancement” pattern: mild homogeneous enhancement in the arterial phase, intensification in the venous phase, and persistent enhancement in the delayed phase, albeit less intense than normal pancreatic parenchyma.^[9]^ MRI findings include hypo-intensity on T1-weighted imaging, iso to mild hyperintensity on T2-weighted imaging, and marked hyperintensity on diffusion-weighted imaging with rare necrotic or cystic changes.^[10]^ A pathognomonic feature is the vascular floating sign, where the tumor encases major vessels (e.g., the celiac trunk or superior mesenteric artery) without luminal invasion or mural irregularity.^[11]^ The diffuse infiltrative subtype (10–15%) presents as diffuse pancreatic enlargement with ill-defined margins, mimicking acute pancreatitis.^[12]^ Enlarged retroperitoneal lymph nodes below the renal vein level strongly suggest PPL.^[13]^ Although not performed in this case, diffusion-weighted imaging is highly valuable for characterizing lymphomatous lesions, which typically show marked restriction. Furthermore, 18F-FDG PET/CT plays a crucial role in initial staging, assessing treatment response, and detecting recurrence by identifying metabolically active disease, and should be considered in the workup of suspected PPL when available.

Definitive diagnosis requires histopathological evaluation via EUS-guided biopsy or laparotomy. EUS-guided biopsy surpasses percutaneous methods by minimizing interference from gastrointestinal gas and adjacent organs, enabling real-time, high-resolution visualization of targeted sampling.^[14]^ This approach achieves a diagnostic accuracy of 85% to 90% while reducing complications (e.g., hemorrhage, pancreatic fistula < 5%).^[15]^ Limitations include operator-dependent sampling errors and insufficient tissue for immunophenotyping. In this case, the initial EUS-FNB yielded only fibrinoid necrosis, likely due to sampling error, which ultimately necessitated surgical resection for definitive diagnosis.

The management of PPL necessitates a multidisciplinary approach that balances oncologic efficacy against procedural risks.^[16]^ The treatment and prognosis of PPL depend on the tumor stage and subtype, with options including chemotherapy, radiotherapy, surgery, or multimodal therapy.^[1]^ The R-CHOP regimen remains the first-line therapy for CD20 (+) DLBCL, which is the most common PPL subtype. Recent meta-analyses have confirmed that R-CHOP significantly improves outcomes compared to CHOP alone, particularly in elderly patients. For example, the European Lymphoma Network (2023) reported that R-CHOP increased complete remission rates (76% vs 63%), 5-year progression-free survival (54% vs 30%), and overall survival (58% vs 45%), while maintaining a favorable safety profile.^[17]^ Adjuvant radiotherapy is reserved for bulky diseases or high-risk cases, but carries risks of biliary/duodenal strictures.^[18]^ The current NCCN guidelines (2023) recommend its selective use, emphasizing that combined chemoradiotherapy lacks robust evidence in PPL compared with other extranodal lymphomas.^[19]^

The role of surgery in PPL remains controversial, with current guidelines (NCCN, ESMO) prioritizing chemotherapy as the cornerstone treatment.^[20]^ Surgical intervention is generally not recommended unless there is diagnostic uncertainty or complications such as obstruction or bleeding. In this case, a distal pancreatectomy with splenectomy was performed due to inconclusive biopsy results and a high suspicion for malignancy. Emerging evidence suggests potential survival benefits of surgical resection in stage I/II PPL when combined with chemotherapy.^[21]^ However, such findings require validation in larger cohorts. Palliative procedures, such as biliary-enteric anastomosis, may alleviate obstructive jaundice but remain adjunctive to systemic therapy. Pancreaticoduodenectomy, though curative in select cases, it carries significant morbidity (30–40%), including delayed gastric emptying and pancreatic fistula.^[22]^ Technical challenges in large tumors or severe adhesions may delay the initiation of adjuvant therapy and compromise outcomes. Recent meta-analyses caution against routine surgery, favoring chemotherapy-first strategies for comparable survival with reduced complications.^[23]^ This patient achieved sustained remission after distal pancreatectomy and adjuvant chemotherapy, highlighting the role of surgery in diagnostically ambiguous lesions. However, such outcomes must be contextualized within multidisciplinary frameworks and modern therapeutic efficacy.

In summary, PPL is a rare clinical entity that closely mimics pancreatic adenocarcinoma. Its differentiation from adenocarcinoma is crucial, as treatment strategies and prognoses differ significantly. This case highlights the diagnostic challenges of PPL and reinforces the necessity of histopathological confirmation for ambiguous pancreatic lesions. While R-CHOP-based regimens achieve favorable outcomes, the role of surgery in localized disease requires further prospective validation. International collaborative efforts are essential to establish evidence-based guidelines for managing this rare malignancy.

## Author contributions

**Data curation:** Xingdong Xu, Tingting Zhang, Jinyu Liu, Xingzi Li.

**Writing – review & editing:** Gang Wang.
